# Adiponectin gene therapy prevents islet loss after transplantation

**DOI:** 10.1111/jcmm.17515

**Published:** 2022-08-17

**Authors:** Chengshi Wang, Xiaojiong Du, Fudong Fu, Xiaoyu Li, Zhenghao Wang, Ye Zhou, Liping Gou, Wei Li, Juan Li, Jiayi Zhang, Guangneng Liao, Lan Li, Yuan‐Ping Han, Nanwei Tong, Jingping Liu, Younan Chen, Jingqiu Cheng, Qi Cao, Erwin Ilegems, Yanrong Lu, Xiaofeng Zheng, Per‐Olof Berggren

**Affiliations:** ^1^ Key Laboratory of Transplant Engineering and Immunology, Department of Endocrinology and Metabolism, West China Hospital Sichuan University Chengdu China; ^2^ Department of Endocrinology and Metabolism, Center for Diabetes and Metabolism Research, West China Hospital Sichuan University Chengdu China; ^3^ Department of Vascular Surgery, West China Hospital Sichuan University Chengdu China; ^4^ West China Hospital, Institutes for Systems Genetics Sichuan University Chengdu China; ^5^ The Rolf Luft Research Center for Diabetes and Endocrinology Karolinska Institutet Stockholm Sweden; ^6^ Key Laboratory of Bio‐Resource and Eco‐Environment of Ministry of Education, The Center for Growth, Metabolism and Aging, The College of Life Sciences Sichuan University Chengdu China; ^7^ Centre for Transplant and Renal Research, Westmead Institute for Medical Research The University of Sydney Sydney New South Wales Australia

**Keywords:** Adiponectin, gene therapy, hypoxia/reoxygenation injury, inflammation, islet transplantation, oxidative stress

## Abstract

Significant pancreatic islet dysfunction and loss shortly after transplantation to the liver limit the widespread implementation of this procedure in the clinic. Nonimmune factors such as reactive oxygen species and inflammation have been considered as the primary driving force for graft failure. The adipokine adiponectin plays potent roles against inflammation and oxidative stress. Previous studies have demonstrated that systemic administration of adiponectin significantly prevented islet loss and enhanced islet function at post‐transplantation period. In vitro studies indicate that adiponectin protects islets from hypoxia/reoxygenation injury, oxidative stress as well as TNF‐α‐induced injury. By applying adenovirus mediated transfection, we now engineered islet cells to express exogenous *adiponectin* gene prior to islet transplantation. Adenovirus‐mediated adiponectin transfer to a syngeneic suboptimal islet graft transplanted under kidney capsule markedly prevented inflammation, preserved islet graft mass and improved islet transplant outcomes. These results suggest that adenovirus‐mediated *adiponectin* gene therapy would be a beneficial clinical engineering approach for islet preservation in islet transplantation.

## 1 INTRODUCTION

Pancreatic islet transplantation has been considered a promising treatment strategy for type 1 diabetes mellitus (T1DM). However, significant islet loss and dysfunction shortly after transplantation to the liver limits its widespread implementation in the clinic.^1^ Up to 60% of the transplanted islets were destroyed even in immunodeficient and syngeneic transplantation models, highlighting the critical roles of nonimmune factors in determining islet transplant outcomes.^2^, ^3^ Reactive oxygen species and inflammation resulting from islet isolation and ischaemia–reperfusion injury (IRI) have been considered as the primary cause of graft loss in the early post‐transplantation period.^4^, ^5^ It is therefore of importance to equip islets with anti‐oxidative and anti‐inflammatory properties prior to transplantation.

Adiponectin is an adipokine mainly produced and secreted by adipocytes. The beneficial roles of adiponectin against inflammation and oxidative stress have been demonstrated in numerous organs and cells.^6^, ^7^, ^8^, ^9^ Additionally, adiponectin has also been shown to promote angiogenesis via activation of VEGF and AMPK pathways.^10^ Therefore, adiponectin may serve as a potential endogenous factor improving islet transplant outcomes. Interestingly, adiponectin has previously been shown to prevent islet ischaemia–reperfusion injury by negatively regulating the COX2–TNF‐α–NF‐κB‐dependent signalling pathway.^11^

Gene therapy presents a novel strategy to genetically modify islet cells ex vivo prior to transplantation and improve islet transplant outcomes. Numerous studies have demonstrated that genetic modification of islets with immunomodulatory, anti‐apoptotic or angiogenic genes can significantly improve the islet engraftment.^12^, ^13^ Adenovirus (Ad) vector‐based gene transfer system has many favourable features such as high infection efficiency, high capacity to accept large amounts of additional DNA and the ability of infecting both replicating and quiescent cell populations, which makes it superior to other vectors.^14^ Therefore, it has been widely used in both preclinical and clinical studies and accounts for >20% of all gene therapy trials.^15^

In this study, a suboptimal mass of syngeneic islets was genetically modified to express exogenous *adiponectin* gene via adenovirus‐mediated gene transfer and transplanted into Balb/c mice under the kidney capsule. Our results demonstrate that adenovirus‐mediated *adiponectin* gene therapy prevents islet loss, which correlates with improved glycaemic control in diabetic mice.

## 2 MATERIALS AND METHODS

### 2.1 Animals

Adult male Balb/c mice (10‐week‐old) were purchased from Chengdu Dashuo Biological Technology Co., Ltd. The animals were housed in a room with controlled temperature (20–22°C), humidity (40%–60%) and 12‐h light/dark cycle. The animals were fed with standard chow and sterile water ad libitum. All animal studies and experimental protocols were approved and reviewed by the Animal Ethics Committee of the Sichuan University, which are consistent with the National Institutes of Health Guide for the Care and Use of Laboratory Animals.

### 2.2 Mouse islet isolation and culture

Islets were isolated from male Balb/c mice using established methods.^16^ Briefly, the pancreas was distended with 3 ml of 0.5 mg/ml Liberase TL (Roche) solution and digested for 10 min at 37°C in a water bath. Islets were purified by density gradient centrifugation, collected and washed with Hank's solution containing 1% BSA (Roche). Then, islets were stained with dithizone (Sigma) and counted under the microscope. Islets were cultured in RPMI‐1640 medium (Gibco, Invitrogen) supplemented with 10% foetal bovine serum (FBS, Thermo Fisher Scientific), 100 U/ml penicillin and 100 U/ml streptomycin.

### 2.3 Adenovirus‐mediated *adiponectin* gene transfer to islet cells

Purified islets were treated with trypsin–ethylenediaminetetraacetic acid (trypsin–EDTA; Invitrogen) and dispersed into single cells. Single islet cells were infected with Ad‐adiponectin‐GFP/Ad‐GFP (Shanghai Genechem Co., LTD) at a multiplicity of infection (MOI) of 100 for 2 h. 72 h after viral infection, cells were stained with Hoechst 33342 (Thermo Fisher Scientific Inc.) and subjected to fluorescence imaging of GFP and nuclei (Leica SP8 system). RT‐qPCR and Western blot analysis were performed to identify the expression of adiponectin in the islet cells at 0, 24, 36, 48 and 72 h after viral infection.

### 2.4 Aggregation of dissociated single islet cells into islet‐like cell clusters

Prior to functional readout and islet transplantation, the dissociated single islet cells were aggregated as cell clusters as previously described.^17^ Briefly, the dissociated islet cells were washed twice with HBSS after viral infection. The islet cells (2 × 10^5^ cells/dish) were then seeded into a non‐adhesive 35 mm culture dish (Corning) and cultured in RPMI‐1640 medium for 4 days until islet‐like cell clusters were formed.

### 2.5 Glucose‐stimulated insulin secretion (GSIS) assay

The glucose‐stimulated insulin secretion assay was performed using established methods.^18^ Briefly, ad‐transduced islet‐like cell clusters were preincubated for 1 h in HEPES‐balanced Krebs‐Ringer bicarbonate buffer (119 mM NaCl, 4.74 mM KCl, 2.54 mM CaCl2, 1.19 mM MgCl2, 1.19 mM KH2PO4, 25 mM NaHCO3, 10 mM HEPES and 0.5% BSA, pH 7.4) containing 2.8 mM glucose. The cells were then incubated in the same buffer containing 2.8 mM (basal concentration) or 20 mM glucose (stimulatory concentration) for 1 h at 37°C. At the end of the glucose challenge, the insulin levels in the supernatant were measured by an enzyme‐linked immunosorbent assay (ELISA, Linco Research) and normalized to total protein of the cells.

### 2.6 In vitro hypoxia–reoxygenation injury model and oxidative damage model

To induce hypoxia‐reoxygenation (H/R) injury, the islet‐like cell clusters were subjected to hypoxic conditions (1% O2, 5% CO_2_ and 94% N_2_) for 8 h, followed by reoxygenation (21% O2, 5% CO_2_, 74% N_2_) for 16 h. The islet‐like cell clusters cultured for the same duration under ordinary conditions were considered as control. To induce oxidative damage, the islet‐like cell clusters were exposed to culture mediums containing 300 μM H_2_O_2_ for 2 h.

### 2.7 In vitro inflammation model induced by TNF‐α

To mimic islet inflammation after transplantation, islet‐like cell clusters were stimulated with TNF‐α (1000 U/ml, R&D Systems) for 48 h. Islet‐like clusters without any treatment were used as control.

### 2.8 Measurement of cell death by propidium iodide staining and flow cytometry

The adenovirus‐transduced islet‐like cell clusters were dispersed into single cells prior to the measurement of cell death. For propidium iodide (PI) staining, cells were incubated with 10 μg/ml PI in medium for 1 h at 37°C. Dead cells were measured immediately by FACSCalibur flow cytometer and analysed using FlowJo Software Version 10.

### 2.9 ELISA analysis

Seven days post‐transplantation, blood samples were collected from the tail vein, and the levels of insulin, TNF‐α, IL‐2 and IFN‐γ in serum were measured using different ELISA kits (Cusabio Biotech Co., LTD), according to the manufacturer's instructions. All data were measured using a plate reader (SYNERGY H1, BioTek).

### 2.10 Quantitative real‐time polymerase chain reaction (RT‐qPCR)

Total RNA was isolated from cells and islet grafts using Trizol (Gibco, Life Technologies), following the manufacturer's instructions. Isolated RNA was quantified using the NanoDrop 2000 spectrophotometer (Thermo Fisher Scientific Inc). RNA was reverse transcribed to cDNA using the iScript cDNA Synthesis kit (Bio‐Rad). The primer sequences are listed in Table S1. The qPCR analysis was performed on a CFX96 Real‐Time PCR Detection System (Bio‐Rad) with SYBR green supermix (SsoFast EvaGreen, Bio‐Rad). The relative change in the expression of target genes mRNA was calculated using the 2^‐ΔΔ^CT method.

### 2.11 Western blotting analysis

Cells were homogenized in lysis buffer containing phenylmethanesulphonyl fluoride (PMSF). Protein concentrations were measured using the bicinchoninic acid (BCA) protein assay kit (Beyotime Biotechnology). Equal amounts of protein were subjected to sodium dodecyl sulphate‐polyacrylamide gel electrophoresis (SDS‐PAGE). Resolved proteins were transferred to a polyvinylidene difluoride membrane (0.22 μm PVDF, Merck Millipore). The membrane was incubated with 5% nonfat milk for 1 h for blocking. Next, the membrane was probed with the primary antibodies against Adiponectin (1:500, Abcam), PGE2 (1:500, Abcam), COX2 (1:500, Abcam), p‐NF‐κB (1:500, Abcam), NF‐κB (1:500, Abcam) or β‐Actin (1:5000, Abcam) overnight at 4°C. The membrane was then incubated with the horseradish peroxidase (HRP)‐conjugated secondary antibodies (1:2000, Santa Cruz Biotechnology, Inc.). The protein bands were visualized using the enhanced chemiluminescence (ECL) reagents in the Molecular Imager Gel Doc XR System (Bio‐Rad). The blots were subjected to densitometric analysis using the Image J software (NIH). The protein expression was normalized to the expression of β‐actin.

### 2.12 Syngeneic islet transplantation

Diabetic Balb/c mice (22‐25 g, male) were used as recipients. Diabetes was induced in mice by single intraperitoneal injection of streptozotocin (STZ, 200 mg/kg, Sigma‐Aldrich) 10 days prior to transplantation. One week following STZ injection, animals with non‐fasting blood glucose levels >250 mg/dl were considered as diabetic. The vital signs of the animals were monitored daily and none of the animals exhibited severe signs of illness or died due to the experimental treatment. Diabetic Balb/c mice were randomly divided into two groups and transplanted under the renal capsule with suboptimal amount of Balb/c islet‐like cell clusters (150 islet equivalents, IEQ) which were transduced with Ad‐Adiponectin‐GFP (Ad‐Adi, *n* = 8) or Ad‐GFP (Ad‐GFP, *n* = 8) using the established procedure.

### 2.13 Evaluation of islet graft function

Recipients of suboptimal mass syngeneic islets had their blood glucose monitored approximately every other day for 30 days. At Day 30, nephrectomy of the grafted kidney was performed to confirm graft dependent efficacy, when animals returned to hyperglycaemia (≥16.5 mmol/L). To evaluate the islet graft function in vivo, intraperitoneal glucose tolerance test (IPGTT) and glucose stimulated c‐peptide test were performed at postoperative Day 30. Briefly, after 16 h of fasting, mice were intraperitoneally injected with glucose solution (2 g/kg body weight). Blood glucose measurements were taken at 0, 30,60, 90 and 120 min after glucose injection. The serum C‐peptide levels were measured at 0 and 30 min after glucose injection.

### 2.14 Histological examination

Histological examination was performed as previously described.^19^ Renal tissues containing islet grafts were cut and fixed with 10% formalin at room temperature for 24 h. Consecutive sections (5 um thick) of paraffin‐embedded renal tissues containing islet grafts were cut and subjected to immunohistochemical (IHC) staining. For IHC staining, sections were blocked with 1% BSA, and incubated with diluted primary antibodies including rabbit anti‐insulin (Abcam), then incubated with horseradish peroxidase (HRP)‐conjugated secondary antibody (DAKO) and finally stained with 3,3′‐diaminobenzidine (DAB) substrate and haematoxylin. Immunofluorescence (IF) was performed with goat anti‐mice insulin (Abcam) and rabbit anti‐mice CD31 (Abcam). The images of stained sections were acquired by fluorescence microscope (Carl Zeiss), and insulin and CD31 positive area were quantified using Image J software.

### 2.15 Statistical analysis

Descriptive statistics were analysed by Graphpad Prism 8 and presented as mean ± standard error of mean (SEM). Outliers were identified using Grubbs' test. Data were analysed using the Kolmogorov–Smirnov test to confirm the normal distribution. Two‐sided student's *t* test was applied for comparisons between two groups. One‐way analysis of variance (anova) followed by Tukey's ad hoc test was used for multiple comparisons. All data were analysed using the two‐tailed test. The difference was considered statistically significant when the *p* < 0.05. All measures were taken from distinct samples, and the sample sizes are presented in figure legends. All the in vitro experiments were performed at least three times independently.

## 3 RESULTS

### 3.1 Adenovirus‐mediated adiponectin transduction has no negative impact on islet cell function or viability

**FIGURE 1:**
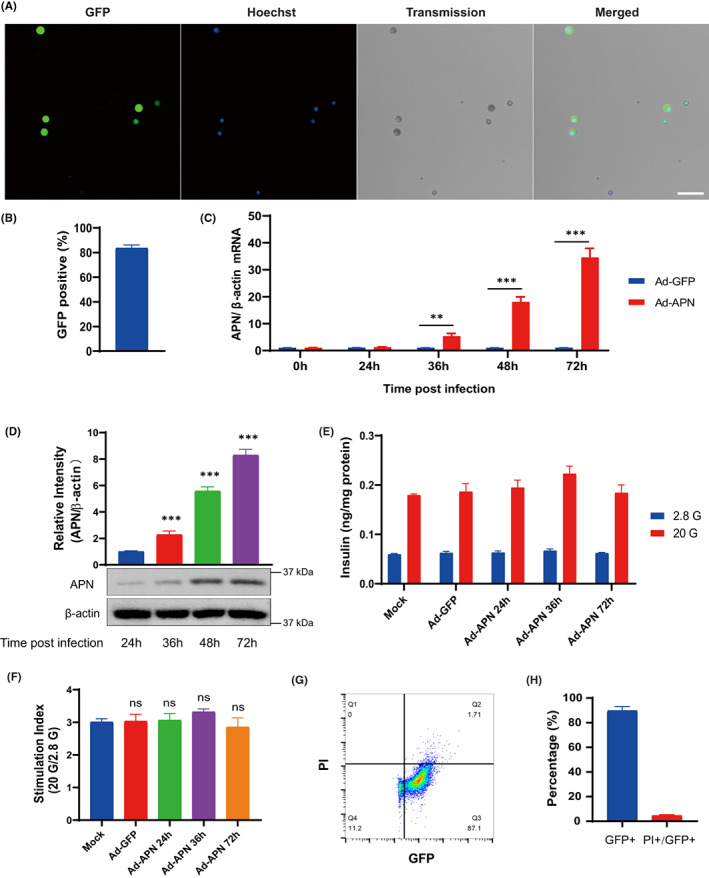
Adenovirus‐mediated adiponectin transduction is not toxic to islet cells. (A) Typical fluorescence microphotographs of primary islet cells infected with Ad‐Adiponectin‐GFP (Ad‐APN, MOI = 100) at 72 h post‐transfection. Scale bar = 50 μm. (B) Quantification of the percentage of GFP positive cells among islet cells infected with Ad‐APN for 72 h. *n* > 200 cells from three independent pancreatic islet isolations were analysed. (C) qPCR analysis of adiponectin mRNA levels in islet cells infected with Ad‐GFP or Ad‐APN at indicated time post‐transfection (*n* = 5). (D) Western blot analysis of the expression levels of adiponectin in islet cells infected with Ad‐APN at indicated time points post‐transfection (*n* = 4). (E) GSIS of islet cells under indicated treatments (*n* = 4). (F) Stimulation index calculated by the ratio of insulin secretion at 20 mmol/L glucose (20 G) to insulin secretion at 2.8 mmol/L glucose (2.8 G) in islet cells with indicated treatments (*n* = 4). (G and H) Flow cytometry analysis and quantification of adiponectin gene transduction efficiency (percentage of GFP+ cells) and the apoptotic rate (percentage of PI+ cells among GFP+ cells) of islet cells infected with Ad‐APN at 72 h post‐transfection (*n* = 4). Values are mean ± SEM; ns, not significant; **p < 0.01; ***p < 0.001.

We first evaluated whether exogenous adiponectin expression would be toxic and interfere with islet cell function. To improve transduction efficiency, freshly isolated islets were dispersed into single cells and exposed to adenovirus carrying adiponectin‐GFP/GFP. Viral infection rate was examined in the Ad‐transduced islet cells with fluorescence microscopy and GFP positive cells were around 83.7% at 72 h after transduction (Figure 1A,B). The expression levels of adiponectin were further confirmed with RT‐qPCR and Western blot in Ad‐transduced islet cells at 24, 36, 48 and 72 h after transduction. Both mRNA and protein levels of adiponectin were significantly increased in Ad‐adiponectin‐GFP‐transduced islet cells from 36 to 72 h post‐transduction in a time‐dependent manner, while adiponectin was barely detected in Ad‐GFP‐transduced islet cells (Figure 1C,D). To examine the impact of adiponectin expression on islet cell function, GSIS assay was performed in Ad‐adiponectin‐GFP‐transduced islet cells at 24, 36 and 72 h post‐transduction and compared with non‐transduced or Ad‐GFP‐transduced islet cells. No statistically significant differences were observed among the different groups (Figure 1E,F). To investigate if adiponectin expression induced islet cell death, PI staining was performed in Ad‐adiponectin‐GFP‐transduced islet cells at 72 h post‐transduction, followed by flow cytometry analysis. Around 50,000 cells were analysed in each experiment and around 90% of the cells were GFP positive (Figure 1G,H), which was consistent with our data from fluorescence imaging (Figure 1A,B). Less than 5% PI positive cells were found in the GFP positive cell population (Figure 1G,H). Taken together, the islet cells can be effectively transduced by Ad‐adiponectin‐GFP/Ad‐GFP without negatively affecting islet cell function or viability.

### 3.2 Adiponectin transduction protects islet cells from hypoxia/reoxygenation injury

**FIGURE 2:**
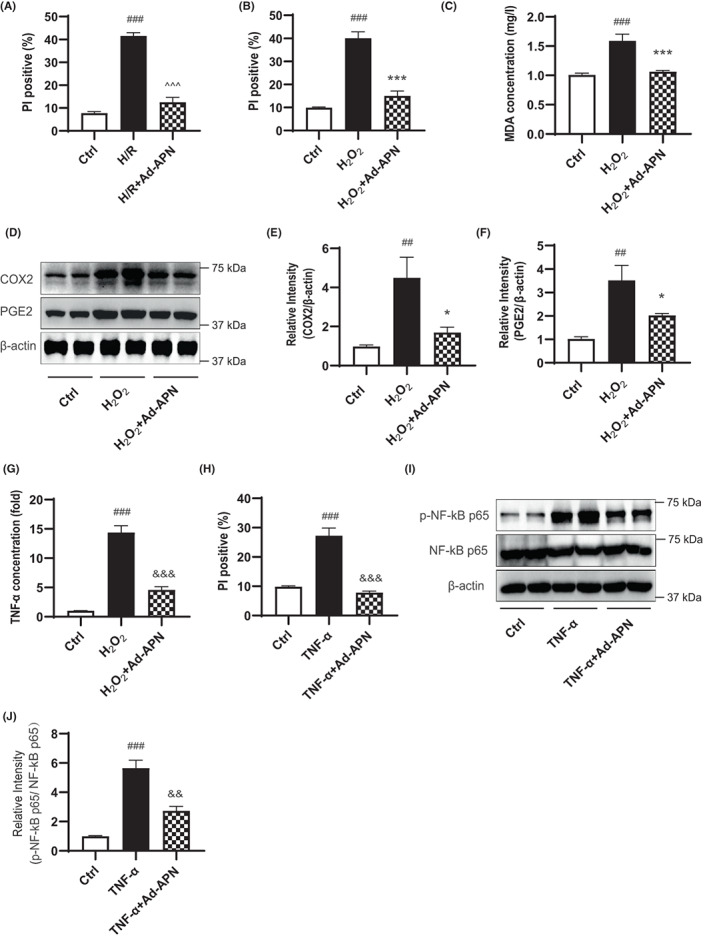
Islet cells expressing exogenous adiponectin gene are resistant to hypoxia/reoxygenation injury. (A and B) Flow cytometry analysis of the apoptotic (PI positive cells) rate of islet cells with indicated treatments (*n* = 5). (C) Measurement of MDA concentrations in islet cells with indicated treatments (*n* = 5). (D–F) Western blot analysis and quantification of the expression levels of COX2 and PGE2 in islet cells with indicated treatments (*n* = 5). (G) Measurement of TNF‐α levels in the culture medium of islet cells with indicated treatments (*n* = 5). (H) Flow cytometry analysis of the apoptotic (PI positive cells) rate of islet cells with indicated treatments (*n* = 5). (I and J) Western blot analysis and quantification of the expression levels of p‐NF‐kB p65 and NF‐kB p65 in islet cells with indicated treatments (*n* = 4). Values are mean ± SEM; ^##^ *p* < 0.01, ^###^ *p* < 0.001 versus Ctrl group; ^^^^^ *p* < 0.001 versus H/R group; *p < 0.05, ***p < 0.001 versus H_2_O_2_ group; ^&&^ *p* < 0.01, ^&&&^ *p* < 0.001 versus TNF‐α group.

Hypoxia/reoxygenation (H/R) injury is the key factor associated with post‐transplant islet graft failure and oxidative stress plays an important role in H/R‐induced cell injury. We investigated the impact of adiponectin expression on hypoxia/reoxygenation injury and oxidative stress. Reoxygenation of hypoxic islet cells induced more than 40% cell death, whereas *adiponectin* gene transfer rescued H/R‐induced islet cell death to 12% (Figure 2A). Next, we used hydrogen peroxide (H_2_O_2_) to trigger oxidative stress in islet cells, simulating in vivo pathological conditions. H_2_O_2_ exposure induced cell death in 40% of the islet cells, and adiponectin gene transfer significantly reversed this effect (Figure 2B). As predicted, H_2_O_2_ exposure increased the levels of the oxidative stress maker malondialdehyde (MDA) by approximate 1.5‐fold, whereas H_2_O_2_‐induced MDA production was significantly decreased in adiponectin‐transduced islets (Figure 2C). In addition, adiponectin gene transfer significantly decreased H_2_O_2_‐induced expression of the inflammatory molecules COX2, PGE2 and TNF‐α (Figure 2D–G).

TNF‐α plays a key role in mediating ischaemia–reperfusion injury and acts as a cytokine marker during organ rejection.^20^, ^21^ To investigate the impact of adiponectin on TNF‐α‐induced injury, islet‐like cell clusters were treated with 1000 U/mL TNF‐α for 48 h and cell viability were evaluated. Our data showed that adiponectin gene transfer significantly prevented TNF‐α‐induced islet cell death (Figure 2H). NF‐κB plays an important role in the transcriptional regulation stimulated by TNFα.^22^, ^23^ Next, we examined the effect of adiponectin on TNFα‐induced NF‐κB activation. TNF‐α‐induced a 5.5‐fold increase in p‐NF‐κB p65 protein levels, whereas adiponectin gene transfer significantly decreased this effect (Figure 2I,J).

Together, these results indicate that adenovirus‐mediated adiponectin transduction could protect islet cells from hypoxia/reoxygenation injury, oxidative stress and TNF‐α‐induced injury.

### 3.3 Adiponectin transduction improves islet graft function and survival

**FIGURE 3:**
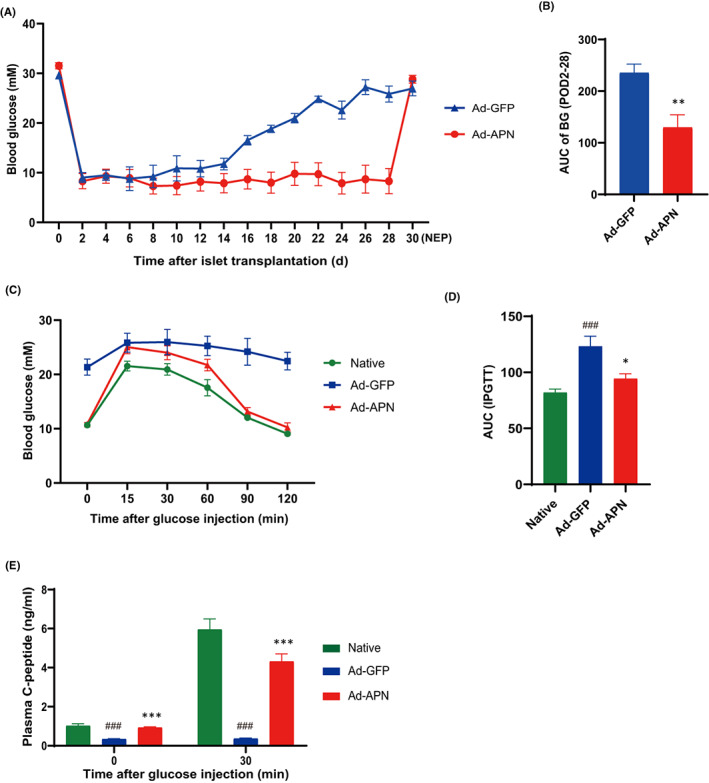
Adiponectin transduction improves islet transplant outcomes. (A) Monitoring of blood glucose levels after islet transplantation until nephrectomy (NEP) was performed at post‐transplant day (POD) 30 (*n* = 6). (B) AUC of blood glucose levels from POD2 to POD28 (*n* = 6). (C and D) IPGTT on native Balb/c mice (Ctrl), diabetic Balb/c mice transplanted with Ad‐GFP islets (Ad‐GFP) or Ad‐APN islets (Ad‐APN) and AUC of blood glucose levels (*n* = 6). (E) Plasma C‐peptide levels of indicated animals at 0 and 30 min after glucose injection (*n* = 6). Values are mean ± SEM; ^###^ *p* < 0.001 versus Ctrl group; *p < 0.001, ***p < 0.001 versus Ad‐GFP group.

Next, we investigated if expression of adiponectin could improve islet survival and function after transplantation. A suboptimal number of syngeneic Ad‐adiponectin‐GFP/Ad‐GFP‐transduced islet‐like cell clusters (150 IEQ/recipient) were transplanted under the kidney capsule in STZ‐induced diabetic Balb/c mice, and metabolic parameters of the animals were analysed. Our results show that Ad‐GFP‐transduced islet cells could only rescue hyperglycaemia of the diabetic animals at the early‐stage post‐transplantation and failed to maintain their normal glycaemia after 2 weeks of transplantation. On the contrary, Ad‐adiponectin‐GFP‐transduced islet cells significantly extended glycaemia control over 30 days until nephrectomy was performed (Figure 3A). Area under curve (AUC) of blood glucose (BG) at post‐transplant day (POD) 2–28 was significantly reduced in the Ad‐adiponectin‐GFP‐transduced group compared with that of Ad‐GFP‐transduced control group (Figure 3B). Interestingly, the Ad‐adiponectin‐GFP‐transduced group became hyperglycaemic again after nephrectomy at POD 30, indicating that the ameliorated glucose control indeed depended on the transplanted islet grafts (Figure 3A). At POD 30, glucose tolerance in the Ad‐adiponectin‐GFP‐transduced group was significantly improved compared with the Ad‐GFP‐transduced group (Figure 3C,D). Moreover, the fasting and glucose‐stimulated serum C‐peptide levels of the Ad‐adiponectin‐GFP‐transduced group were dramatically higher than those of Ad‐GFP‐transduced group (Figure 3E). Our data indicate that adenovirus‐mediated adiponectin transduction of islet cells could significantly improve the outcomes of syngeneic renal subcapsular islet transplantation.

### 3.4 Adiponectin transduction preserves islet cell mass

**FIGURE 4:**
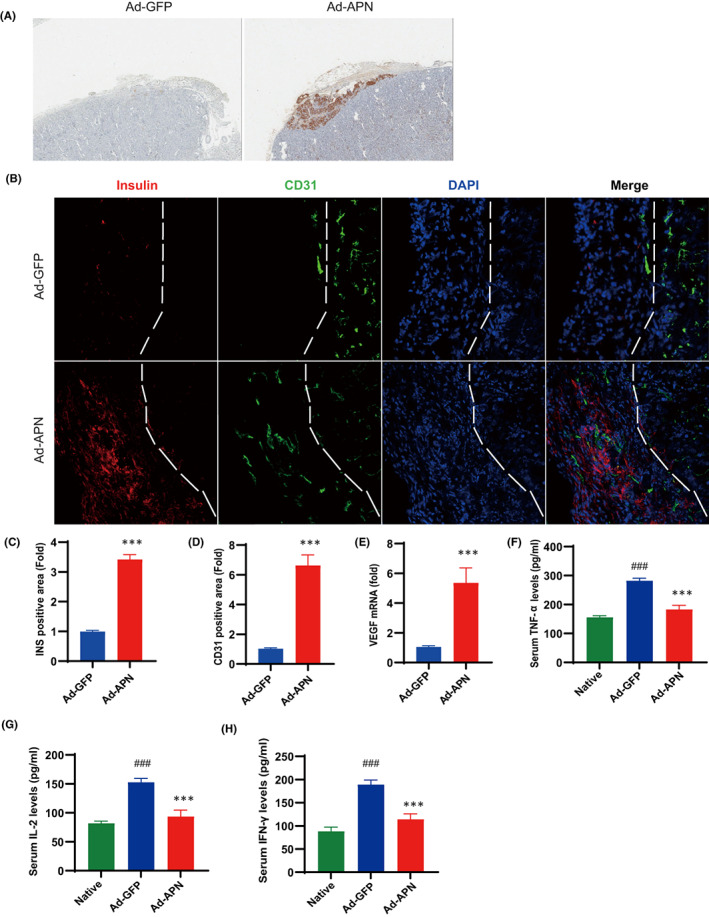
Adiponectin transduction reduces inflammatory responses and prevents islet graft loss. (A) Immunohistochemical analysis of insulin at implantation sites of the mouse at POD 30. (B) Fluorescent staining of insulin (red) and CD31 (green) in implantation sites of the mouse at POD 30. DAPI was used to stain the cell nucleus (blue). (C and D) Quantification of insulin and CD31 positive areas. (E) qPCR analysis of VEGF mRNA levels in islet grafts (*n* = 5). (F–H) Measurement of the levels of serum TNF‐α, IL‐2 and IFN‐γ (*n* = 5). Values are mean ± SEM; ^###^ *p* < 0.001 versus Native group; ***p < 0.001 versus Ad‐GFP group.

To access islet graft morphology, grafts were dissociated from recipient mice at POD 30 and routinely processed for histology. The Ad‐adiponectin‐GFP‐transduced group showed well‐preserved islets without significant evidence of degeneration or destruction, whereas islet cells were barely detected in the Ad‐GFP‐transduced control group (Figure 4A). Number of insulin positive areas in the Ad‐adiponectin‐GFP‐transduced group was significantly higher than that in the Ad‐GFP‐transduced control group (Figure 4B,C). To evaluate the impact of adiponectin on islet vascularization, the endothelial cell maker CD31 was stained and quantified. A more pronounced CD31 staining was evident in the Ad‐adiponectin‐GFP‐transduced group compared with its control (Figure 4B,D). Separately, the expression levels of the angiogenic factor VEGF‐α were analysed in dissociated islet grafts by RT‐qPCR. VEGF‐α expression in the Ad‐adiponectin‐GFP‐transduced group was significantly higher than that of the Ad‐GFP‐transduced control group (Figure 4E). Considering the anti‐inflammatory property of adiponectin, we also investigated its roles on the early post‐transplant inflammatory response. At POD 7, the levels of TNF‐α, IFN‐γ and IL‐2 were analysed in serum by ELISA. The levels of TNF‐α, IL‐2 and IFN‐γ were significantly lower in the Ad‐adiponectin‐GFP‐transduced group than the Ad‐GFP‐transduced control group (Figure 4F–H).

## 4 DISCUSSION

The success of islet transplantation is limited by the multiple post‐transplant stresses, including inflammatory reactions and oxidative stress, that compromise islet cell function and survival. Strategies to promote islet revascularization and engraftment or prevent islet graft loss are required to improve the outcome of islet transplantation. Gene therapy is a novel and powerful therapeutic approach that may be successfully applied also in islet transplantation. Isolated islets can be genetically modified ex vivo prior to transplantation, avoiding potential systemic side effects. In this context, delivery of immunomodulatory genes, anti‐apoptotic genes or angiogenic genes significantly prolonged islet graft survival and improved islet transplant outcomes.^24^, ^25^, ^26^ Adiponectin is an adipocyte‐derived hormone that regulates metabolism of lipids and glucose. The anti‐inflammatory, anti‐oxidative stress and vascular protective properties of adiponectin make it a potential gene therapy candidate in islet transplantation. In this study, we investigated if genetic engineering of islets with adiponectin improves outcome of islet transplantation.

The efficiency of available methodologies to modulate gene expression in the context of intact pancreatic islets is suboptimal, due to their three‐dimensional structure composed of a large number of cells. To improve gene transfer efficiency, islets were dispersed into single cells prior to adenovirus infection. The dissociated islet cells were then aggregated into islet‐like cell clusters for the functional reconstitution and transplantation. We reached more than 80% of infection efficiency and the expression levels of adiponectin was dramatically elevated after infection without introducing any negative effects on islet cell function and survival.

A growing body of evidence suggests that adiponectin plays anti‐inflammatory roles under certain physiological and pathological conditions. Decreased serum levels of adiponectin are closely associated with elevated inflammation in metabolic disorders such as obesity and type 2 diabetes.^27^, ^28^ Adiponectin has been shown to interfere with NF‐κB, the pivotal mediator of inflammatory responses, thereby inhibit pro‐inflammatory cytokine production in various tissues.^29^, ^30^ It also affects macrophage activity. In addition to suppressing mature macrophage functions, adiponectin promotes cellular differentiation of monocytes into anti‐inflammatory M2 macrophages and suppresses their differentiation into proinflammatory M1 macrophages.^31^, ^32^ Our in vitro studies showed that TNF‐α‐induced cell death and dysfunction, while NF‐kB activation were significantly rescued in adiponectin‐transduced islet cells. Moreover, adiponectin gene transfer significantly reduced levels of peripheral blood inflammatory cytokines (TNF‐α, IFN‐r and IL‐2) at the early post‐transplant period.

Oxidative stress plays a key role in ischaemia–reperfusion injury during islet isolation and transplantation. Adiponectin has been demonstrated to protect against oxidative stress‐induced damage in various tissues. Adiponectin may protect the myocardium from ischaemia/reperfusion injury by suppressing inducible nitric oxide synthase and NADPH oxidase expression or via AMP‐activated protein kinase (AMPK)‐ and cyclooxygenase (COX)‐2‐dependent mechanisms.^33^, ^34^ Its protective effects on endothelial cells partly depend on enhancing endothelial nitric oxide synthase (eNOS), which in turn increases the production of nitric oxide (NO).^35^ Adiponectin has also been shown to up‐regulate the expression of antioxidant enzyme superoxide dismutase (SOD2) and reduce production of reactive oxygen species (ROS).^36^, ^37^ In the current study, adiponectin gene transfer significantly prevented H/R injury‐mediated islet cell death and rescued H/R injury‐mediated islet cell dysfunction. Furthermore, adiponectin gene transfer attenuated islet cell death, improved islet cell function and reduced expression of oxidative stress marker malondialdehyde (MDA) under H_2_O_2_ treatment.

Beside its anti‐inflammatory and anti‐oxidative stress properties, adiponectin also exerts beneficial actions on vasculature. Adiponectin‐KO mice exhibit reduced endothelium‐dependent vasodilation and impaired angiogenesis in response to tissue ischaemia, whereas overexpression of adiponectin accelerates angiogenic repair of ischaemic tissues in an AMP‐dependent manner.^38^, ^39^ Adiponectin has also been shown to induce VEGF‐A expression and promote angiogenesis.^40^ In addition, adiponectin stimulates endothelial cell differentiation into capillary‐like structures by promoting cross‐talk between AMP‐activated protein kinase and Akt signalling.^41^ Furthermore, adiponectin protects against endothelial cell injury by activation of nitric oxide synthase (eNOS) and AMPK signalling.^42^, ^43^ In the present study, we showed that adiponectin gene transfer significantly induced VEGF‐α expression and improved expression of CD31 in the islet graft resulting in more preserved islet cells.

In summary, we have demonstrated that adenovirus‐mediated adiponectin gene therapy could significantly improve islet transplant outcomes. Adiponectin gene transfer promoted angiogenesis of the islet graft and suppressed the inflammatory responses and oxidative stress of the islet cells both in vitro and in vivo. Interventional strategies to improve islet engraftment by adiponectin gene therapy presents as a potential therapeutic approach for successful islet transplantation.

## AUTHOR CONTRIBUTIONS

**chengshi wang:** Conceptualization (equal); data curation (equal); funding acquisition (equal); investigation (equal). **Xiaojiong Du:** Data curation (equal); formal analysis (equal). **Fudong Fu:** Data curation (equal). **Xiaoyu Li:** Data curation (equal). **Zhenghao Wang:** Data curation (equal); formal analysis (equal). **Ye Zhou:** Data curation (equal). **Liping Gou:** Data curation (equal). **Wei Li:** Data curation (equal); formal analysis (equal). **Juan Li:** Data curation (equal). **Jiayi Zhang:** Formal analysis (equal). **Guangneng Liao:** Data curation (equal). **Lan Li:** Data curation (equal). **Yuan‐ping Han:** Writing – review and editing (equal). **Nanwei Tong:** Writing – review and editing (equal). **Jingping Liu:** Writing – review and editing (equal). **Younan Chen:** Writing – review and editing (equal). **Jingqiu Cheng:** Writing – review and editing (equal). **Qi Cao:** Writing – review and editing (equal). **Yanrong Lu:** Conceptualization (equal); funding acquisition (equal); supervision (equal); writing – review and editing (equal). **Xiaofeng Zheng:** Conceptualization (equal); funding acquisition (equal); resources (equal); supervision (equal); writing – original draft (equal). **Per‐Olof Berggren:** Conceptualization (equal); funding acquisition (equal); writing – review and editing (equal). **Erwin Ilegems:** Data curation (equal); formal analysis (equal).

## FUNDING INFORMATION

Program of National Natural Science Foundation of China: 81801589, 82070846. China Postdoctoral Science Foundation: 2018M643487. 1.3.5 project for disciplines of excellence, West China Hospital, Sichuan University: ZYGD18014, ZYGD18017. Center of Excellence‐International Collaboration Initiative Grant of West China Hospital: 139180012. Program for Overseas High‐Level Talents Introduction of Sichuan Province of China: 21RCYJ0046.

## CONFLICT OF INTEREST

P‐OB is founder and CEO of the biotech company Biocrine AB.

## Supporting information


**Table S1** Supplementary table

## Data Availability

The datasets generated during and/or analysed during the current study are available from the corresponding authors on reasonable request.
