# Supplementary material for: Adiponectin gene therapy prevents islet loss after transplantation
Source: J Cell Mol Med. 2022 Aug 17;26(18):4847–58. doi: 10.1111/jcmm.17515 (PMC9465193; doi:10.1111/jcmm.17515)
Supplement: Supplementary file 1 — Table S1 Supplementary table [file JCMM-26-4847-s001.docx]

| Oligonucleotides | |
| --- | --- |
| β-actin forward | CGTGCGTGACATCAAAGAGAA |
| β-actin reverse | AACCGCTCGTTGCCAATAGT |
| VEGF-α forward | GGAGATCCTTCGAGGAGCACTT |
| VEGF-α reverse | GGCGATTTAGCAGCAGATATAAGAA |
| Adiponectin forward | GCAGAGATGGCACTCCTGGA |
| Adiponectin reverse | CCCTTCAGCTCCTGTCATTCC |

**Table S1**
